# Cutting Carbon with Knife and Bin: The Role of Diet and Food Recycling in the Food System of Ulaanbaatar, Mongolia

**DOI:** 10.3390/foods15111834

**Published:** 2026-05-22

**Authors:** Ankhtuya Bold, Shenghui Cui, Jingjing Yin, Wei Huang, Tselmuun Tsog, Delgerjargal Munkhbaatar, Gerelsukh Batbayar

**Affiliations:** 1 Key Lab of Urban Environment and Health, Institute of Urban Environment, Chinese Academy of Sciences, Xiamen 361021, China; bold@iue.ac.cn (A.B.); jjyin@iue.ac.cn (J.Y.); whuang@iue.ac.cn (W.H.); tselmuun@iue.ac.cn (T.T.); delgerjargal@iue.ac.cn (D.M.); batbayar@iue.ac.cn (G.B.); 2 Institute of Urban Environment, University of Chinese Academy of Sciences, Xiamen 361021, China

**Keywords:** food system, greenhouse gas footprint, Ulaanbaatar, cold region, Mongolia

## Abstract

The global food system (FS) contributes one-third of anthropogenic greenhouse gas (GHG) emissions, yet evidence remains heavily skewed toward temperate-climate cities, leaving cold-climate cities in the Northern Hemisphere understudied. Here, the GHG footprint (GHGF) of the entire FS in Ulaanbaatar, Mongolia, is assessed, accounting for six subsystems spanning food production, processing and storage, retail, transportation, consumption, and food waste. The baseline indicates that the food waste (FW) subsystem dominates the total GHGF (47.13 kg CO_2eq_/kg), contributing 49.3% of overall emissions. It exceeds those from agricultural food production (AFP) (18.5%) and, food & food waste transportation (FFWT) (22.6%). We further evaluate two mitigation scenarios. (1) Under a dietary shift scenario aligned with national dietary guidance, the total GHGF decreases 14.4% while the FW subsystem remains the largest contributor, (2) but the food waste reduction scenario yields a comparable reduction of 15.9%. The findings revealed that decarbonisation lever efficiency can be done through food waste reduction while supporting a circular valorisation strategy, including waste-related GHG liabilities as an energy source in cold-climate cities.

## 1. Introduction

The food system (FS) is a major driver of environmental pressures and accounts for roughly one-third of anthropogenic greenhouse gas (GHG) emissions [1]. The latest studies revealed that across the entire FS, GHG emissions are higher in pre- and post-production [2]. Worldwide, 60–80% of food is consumed in urban areas, where over half of the world’s population lives, but urbanised areas only occupy 1% of the Earth’s surface [3]. Against this backdrop, evaluating the environmental impact, sustainability, and resilience of urban food systems has become increasingly important for improving the efficiency of natural resources use (water, energy, soil, gases, etc.) and safeguarding human well-being. Urbanisation is accelerating worldwide—projected to account for 72.8% of the global population by 2050—and total food demand is expected to rise by 50% between 2013 and 2050 [4]. These trends make the environmental footprint of urban FSs a central research imperative. However, the recent literature highlights a significant deficiency in holistic, science-based carbon footprint assessments that encompass the entire FS life cycle [5,6].

The prevailing body of research remains mainly disjointed, frequently focusing on discrete commodities or individual stages—primarily agricultural production—while systematically neglecting downstream phases such as industrial processing, distribution logistics, retail, end-user consumption, and subsequent waste management [7]. Food waste composition varies strongly with season and income, as shown for similar cold-climate cities [8]. Moreover, agri-food emissions are frequently derived from heterogeneous and non-standardised data sources, reflecting the absence of robust, integrated, system-wide datasets [5]. This lack of comprehensive, interconnected analysis across the whole FS limits the ability to link climate impacts to specific stages and constrains the formulation of evidence-based, targeted mitigation strategies [6]. The knowledge gaps are particularly pronounced for urban FS emissions in the Northern Hemisphere, as most existing studies focus on economically developed and temperate or warm cities. Recent studies indicate that cold and small cities are less studied than large cities in Europe and Oceania [9,10].

Therefore, this study adopts Ulaanbaatar (UB), Mongolia, as a case study. UB is an illustrative cold-climate city where a short growing season can constrain local agricultural supply and increase reliance on imported foods, while extreme winter conditions elevate energy demand for cooking, indoor heating associated with food preparation, and cold-chain storage, potentially shifting emissions toward downstream. In addition, cold-season operational constraints may affect the performance and management of food waste handling and disposal, with implications for the overall mitigation potential of waste-oriented interventions. To address these gaps, this study quantifies the GHG footprint (GHGF) of its entire FS in UB. The analytical framework comprises six subsystems, including agricultural food production (AFP), food and food waste transportation (FFWT), food producers and food storage (FP and FS), food retailers (FR), food consumers (FC), and food waste (FW). The transport stage of food supply chains in low-density cities can contribute up to 30% of the total food system of GHGs [6,11].

**Figure 1:**
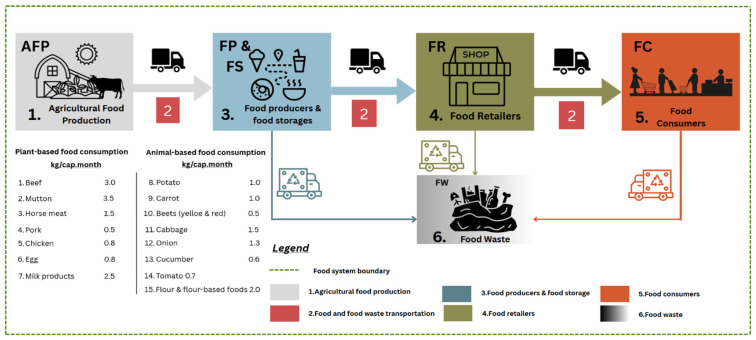
The current food system and the amount of food consumption in UB.

The study aims to answer three key scientific questions: (1) What is the whole GHGF baseline system of UB’s food system, and how is it distributed across the six subsystems? (2) What are the mechanisms and drivers of GHG emissions in a cold-city context? (3) To what extent can different intervention strategies reduce the GHGF of UB’s urban food system? To support these analyses, we compiled a primary database through on-site and real-time measurement of energy use and food-related activities across the UB food system (Figure 1). We employ an integrated methodology combining a Life Cycle Assessment (LCA), scenario analysis (SA) and uncertainty analysis to conduct a comprehensive and policy-relevant evaluation.

## 2. Materials and Methods

### 2.1. Study Boundary and Data Description

**Figure 2:**
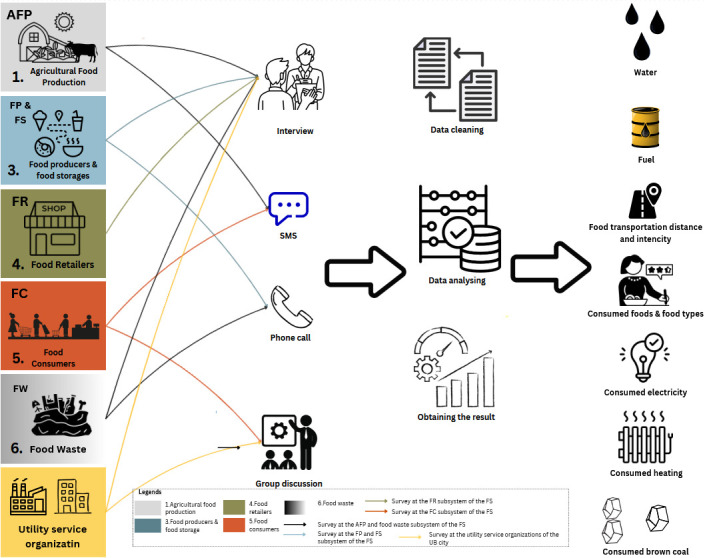
Data collection framework of the field study.

The scope of study covered from the cradle to the grave and includes (i) AFP, (ii) FFWT, (iii) FP and FS, (iv) FR, (v) FC, and (vi) FW, as well as 15 food types with classifications of (a) animal-based and (b) plant-based that represent 78% of the total food dietary consumption by residents under the survey (Figure 1). The study was conducted by (1) face-to face interviews, (2) phone calls, (3) online video calls, and (4) group discussions among the (a) farmers and herders in the AFP and FFWT subsystems; (b) food factories and producers of UB in the FP and FS and FFWT subsystems; (c) open markets and other food markets of UB in the FR and FFWT subsystems; (d) UB households in the FC and FFWT subsystems; (d) government agencies who are responsible for foods, waste management, water supply, energy supply and distribution of UB at the all subsystems; (e) waste truck drivers in the FW and FFWT subsystems; and (f) utility service organisations of UB (Figure 2) for gathering quantitative and qualitative solid data (Appendix B).

After UB residents’ food consumption amounts and types are determined under the survey (Figure 1), the study clarified which data types should be collected in each subsystem. In general, city residents consume a diverse range of 68 food products. Among these, 15 items constitute 74% of the total food consumption. Consequently, these 15 food types were selected for analysis in this study (Figure 1). Under the field survey, a total of 1190 participants were involved (Figure 2), and the study fetched the water and energy (electricity, heating and fuel) consumption, food waste ratio, travel time and distance to purchase food, transportation modes, cooking energy, etc., through all subsystems. Many studies estimated GHG emissions from electricity and heating energy production, following the Tier 1 methodology outlined in the 2019 refinement of the 2006 IPCC Guidelines for National GHG Inventories [12].

To capture seasonal dietary variation, the household survey was administered in two waves: winter (January–February 2023, *n* = 560 households) and summer (July–August 2023, *n* = 559 households). Monthly food consumption quantities were averaged using seasonal weights derived from the Ulaanbaatar Statistical Office’s monthly food supply series (winter weight = 0.45 and summer weight = 0.55, reflecting longer indoor storage in winter).

### 2.2. Data Description

Researchers from the Institute of Urban Environment (Chinese Academy of Sciences) and the Mongolian Academy of Sciences co-developed a culturally tailored dietary questionnaire for Ulaanbaatar, capturing daily, monthly, and seasonal intake. Mixed-mode administration (face-to-face, online calls, and group discussions) ensured cultural comfort. Among 1190 participants, meat and dairy were consumed in equally high amounts year-round, challenging traditional seasonal restrictions. Although lacking formal statistical validation, the questionnaire offers strong content and contextual validity for this urban population.

Subsystem 1. AFP: As we previously demonstrated, GHG emission interventions are sourced from the EcoInvent database/SimaPro 9.5.0 using IPCC 2021 GTP100 V1.02, including the airborne compartment of plant and animal-based foods [13] (Table 1). This study differs from our previous work [13] in three fundamental ways. First, it estimated national-level agricultural production footprints for Mongolia, whereas the current study is city-specific (Ulaanbaatar) and covers all six subsystems from cradle to grave. Second, the previous study mostly used secondary emission factors.

**Table 1:** GHG emission per 1 kg of food [13].

Animal-Based Foods	Total Emission (kg CO_2eq_/kg)	Plant-Based Foods	Total Emission (kg CO_2eq_/kg)
1. Beef	13.34	7. Potato	0.53
2. Sheep meat	5.33	8. Tomato	0.41
3. Pig meat	12.44	9. Cabbage	0.6
4. Chicken meat	2.95	10. Carrot	0.23
5. Hen eggs	0.06	11. Cucumber	0.69
6. Cow milk	2.16	12. Onion	0.48
		13. Wheat	0.82

Subsystem 2. FFWT: Under the field survey of subsystem 2: FFWT, the gasoline consumption of trucks and heavy vehicles across the six subsystems of the FS for food and waste transportation is studied through face-to-face interviews (Figure 2). To be more specific, using the AFP subsystem, a total of 15 farmers and herders were interviewed, and during the field survey, the travel intensity, transportation mode, and gasoline amount in UB were obtained. In terms of subsystem 3, FP and FS, the 25 drivers of trucks and heavy machinery from 13 food factories (flour, milk products, bakery products, meat products, and eggs) participated in the field survey to share how many kilometres they travel to deliver food to the markets and retailers and how much gasoline is used for it within UB. For FC, the (i) travel intensity, (ii) transportation modes and monthly food consumption rates are determined under the survey among 1119 households.

The waste management state companies of UB are involved in the field survey through video call and group discussion to inform the capacity of landfills and the total consumption of gasoline by vehicles and machinery per month. In Equation (1), E_v_ is defined based on “Appendix A. Vehicle fuel consumption per 100 km norm of the Order No. 390 of the Minister of Road and Transportation” [14]. Buses, trucks, and private cars (sedans and SUVs) are included in the vehicle types for food transportation between subsystems. According to the Central Heating Power Plant (CHPP), in subsystems three and four of UB, 273 g of brown coal is utilised for producing 1 kWh of electricity.

(1) $$GHGF_{2}=E_{v}\times \sum_{g}K_{g}\times GWP_{g}$$

where E_v_ is the consumed gasoline by vehicles, g represents gas, and K_CO_2__ = 2.31 kg/L, K_CH_4__ = 0.16 g/L, and K_N_2_O_ = 0.021 g/L are the conversion coefficients [15]. According to the IPCC, GWP_100_ = (1, 28, 273) of CO_2_, CH_4_ and N_2_O to CO_2eq_. GHGF_2_ is the greenhouse gas footprint in the FFWT subsystem per kg of product transport, measured in kg CO_2eq_/kg.

Subsystem 3. FP and FS: At subsystem 3: FP and FS, 13 food producers with their own storage participated. In this subsystem, the GHGF is defined based on its energy and water consumption. Specifically, due to cold-climate conditions in the UB, there are two distinct energy sources: electricity and heating, with brown coal as the main source (Figure 2). Also, water consumption is high in these factories, and water production energy is high in UB as well. Therefore, during the field study, 9 utility service providers (water, electricity, and heating production and supply and distribution state companies) are investigated for the required data for using Equation (2) to estimate the GHGF.

(2) $$GHGF_{3,4,5}=M_{BC}\times \left(E_{kWh}+E_{J}xC_{J\to kWh}\right)\times \sum_{g}E_{g}\times GWP_{g}$$

where *g* represents CO_2_, CH_4_, and N_2_O, and CO_2_ = 0.987 kg, CH_4_ = 9.9 × 10^−6^ kg, and N_2_O = 1.49 × 10^−5^ kg per 1 kg of brown coal burned to produce energy [12]. M_BC_ represents the brown coal mass required to produce 1 kWh of electricity, and in this study, it is 273 g per kWh. C_J-kWh_ is the conversion coefficient between heating and electric energy. It is 2.78 × 10^−7^, which represents the heating energy consumed by the subsystems 3, 4, and 5, and the unit is (joules). KJ/kWh is the conversion coefficient, and here it is 1 J = 2.78 × 10^−7^ kWh/J [16]. According to IPCC, GWP_100_ = (1, 28, 273) of CO_2eq_. GHGF_3,4,5_ is the greenhouse footprint per kg of product, and the measurement unit is kg CO_2eq_/kg.

**Figure 3:**
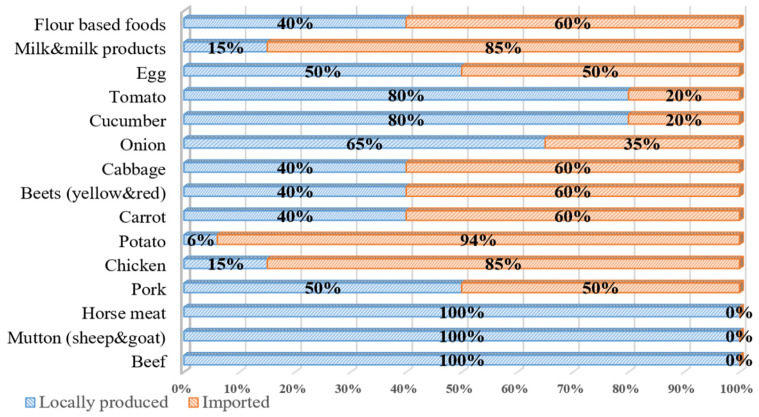
The food source of Ulaanbaatar city (ratio of locally produced and imported foods).

Subsystem 4. FR: Nine food markets and retailers participated in this field survey (Figure 2). The data related to electricity, heating and water consumption are taken into account in the GHGF estimation by using Equation (2). This is not the only data gathered, but also the ratio (Figure 3) of imported foods and locally produced foods. Vendors in marketplaces and shopping centres informed the proportion of food products sold that are imported versus those produced by domestic manufacturers.

Subsystem 5. FC: In the FC subsystem survey, 1119 households are involved (Figure 2) through face-to-face interviews, online calls, and responses to the questionnaire (Appendix 2). Here, food consumption and dietary structure are defined at the household level, which is valuable for identifying the main food types of the FS in UB. Likewise, cooking energy and water utilisation in the kitchen are taken into account to estimate the GHGF by using Equation (2).

Subsystem 6 FW: The amount of food waste is obtained for each survey subsystem, and it is reported that 16% of food waste is produced in the AFP subsystem, while 8% is produced in the FP and FS subsystems. However, in the FR and FC subsystems, it is 17% and 18%, respectively. All food waste is mixed with other waste in each subsystem and transported to the conventional landfills in UB. Therefore, only the energy source of compacting the waste underground at the landfill can be taken into account for the GHGF. Waste management state companies provide the total gasoline consumption of heavy machinery, compactors and vehicles in the landfills of UB. According to the IPCC guidelines, the GHG equivalent in energy from gasoline is 2676 g CO_2_/L, 0.008 g CH_4_/L, 0.033 g N_2_O/L. Using IPCC AR6 100-year GWP, 1 kg of N_2_O = 273 kg CO_2_eq, and 1 kg of CH_4_ = 28 kg CO_2_eq [17].

(3) $$GHGF_{6}=\left(\sum_{i}\alpha_{i}M_{i}\right)x\sum E/\sum Wx\sum_{g}E_{v}^{g}\times GWP^{g}$$

where M_i_ represents the food masses in each subsystem (FP and FS, FR, FC), the measuring unit is kg, and *α_i_* is the food waste ratio of each subsystem (FP and FS = 8%; FR = 17%; FC = 18%). These percentages were defined during the survey among the participants, including households, factories and retailers. E/W is the emission factor, and the measuring unit is L/kg, more specifically, energy per kg of food waste. $E_{v}^{g}$ is the emission factor, g is gas, and the measuring unit is kg CO_2eq_/kg.

## 3. Results

### 3.1. Food Supply Context in UB

Ulaanbaatar (UB) relies on a mixed food supply from local production, domestic sourcing from other provinces, and imports. For vegetables, local production covers only part of the urban demand, and a substantial share is imported from neighbouring countries. Food losses are reported across multiple downstream stages. Limited cold-chain infrastructure during processing and storage contributes to approximately 8% of losses, while handling conditions in retail—dominated by open-air markets—are associated with a further ~17% of losses. At the consumption stage, household food waste is estimated at ~18% of food consumed. Together, these figures indicate that downstream handling and waste management are critical leverage points in UB’s food system.

Under the conducted survey, of the 1148 urban residents approached in Ulaanbaatar, Mongolia, 1119 (food consumers) provided complete and valid responses, yielding a response rate of 97.5%. The sample was predominantly female (775, 69.3%), with a mean age of 36.5 years (SD = 10.8; range 3–77), peaking between the late 20s and early 40s. Household composition revealed a mean of 2.37 adults (SD = 0.98; 57.0% with exactly two adults) and 1.47 children (SD = 1.28; 29.2% with two children), resulting in an average total household size of 3.83 persons (SD = 1.67; media*n* = 4). The majority of respondents (77.6%) held a bachelor’s degree or higher, including 62.6% with a bachelor’s, 14.3% with a master’s, and 0.7% with a PhD. Only 29 cases (2.5%) were excluded due to incomplete data, a proportion unlikely to introduce significant bias. Consequently, the analytic sample represents a relatively well-educated, predominantly female urban population, a demographic profile that should be carefully considered when generalising the study’s dietary and nutritional findings to the broader population of Ulaanbaatar.

Per capita, food consumption in UB is 21.2 kg per month (254.4 kg per year) (Figure 1). Meat products dominated the diet as sheep or goat meat (42.0 kg/year.cap) and beef (36.0 kg/year.cap) were the most significant contributors. Flour-based foods (24.0 kg/year, cap) and milk products (30.0 kg/year, cap) also constituted substantial portions. Concurrently, vegetable intake remained relatively low, but cabbage (18.0 kg/year.cap) and potatoes (12.0 kg/year.cap) were the most consumed. The food supply structure in UB has shifted significantly in recent years toward a greater reliance on imports. Specifically, beef, mutton and horse meat are 100% locally originated, but 80% of tomatoes and cucumbers, 65% of onions, 50% of cabbage, carrots, eggs, and pork are imported from other countries. Locally produced foods are mainly limited to potatoes (94% local), certain vegetables (e.g., 60% of carrots and cabbage), and dairy (85% of milk products) (Figure 3).

### 3.2. Baseline Whole-System GHGF and Subsystem Contributions

**Figure 4:**
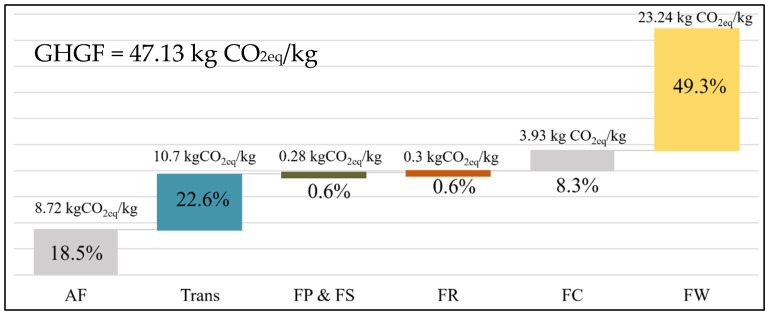
The GHGF of FS in each subsystem in the baseline scenario.

Figure 4 summarises the baseline greenhouse gas footprint (GHGF) across the six subsystems. The estimated total food-system GHGF is 47.13 kg CO_2_-eq per kg of food. Emissions are unevenly distributed across the system: food waste management (FW) is the dominant contributor, accounting for 49.3% (23.24 kg CO_2_-eq/kg). Food and food waste transportation (FFWT) contributes 22.6% (10.66 kg CO_2_-eq/kg), reflecting long supply chains, reliance on diesel freight, and cold-chain logistics [18]. Agricultural food production (AFP) represents 18.5% (8.72 kg CO_2_-eq/kg), consistent with the large role of livestock- and fertiliser-related emissions in agri-food systems [7]. The remaining stages—food processing and storage (FP and FS), food retailers (FR), and food consumption (FC)—account for a smaller share in this assessment (0.6%, 0.6%, and 8.3%, respectively). Overall, the baseline results indicate that UB’s food-system emissions are driven primarily by downstream processes, especially food waste management and transportation, rather than by production alone (Figure 4).

Our scenario analysis shows that a 30% waste reduction (Scenario 2) avoids 9.29 kg CO_2_-eq per kg of food, which is equivalent to taking 34,000 passenger vehicles off the road annually for UB’s total food consumption. To achieve this in practice, we identify three circular economy policies with quantified mitigation potentials:

• Source segregation of organic waste (implemented in 20% of apartments by 2028): 3.1 kg CO_2_-eq/kg reduction.
• Landfill gas capture and flaring (at the three main landfills): 4.8 kg CO_2_-eq/kg reduction.
• Small-scale community composting (targeting ger districts): 1.4 kg CO_2_-eq/kg reduction.

These estimates are derived from the same LCA model but tested separately. Policy makers can prioritise the flaring option because it requires no behavioural change and uses existing heavy machinery.

### 3.3. Scenario 1- Dietary Shift Could Bring Positive Results

**Figure 5:**
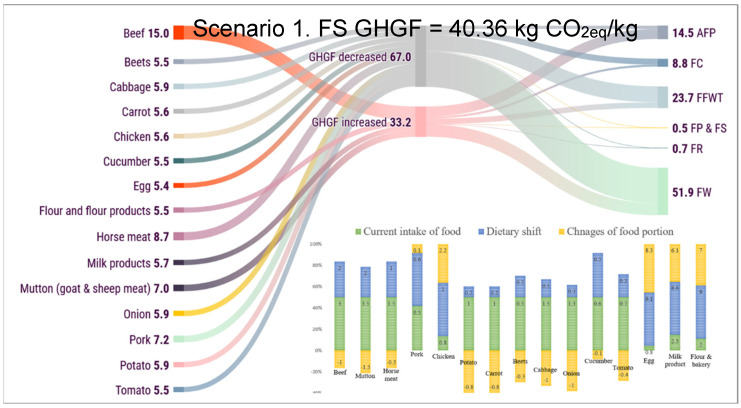
The GHGF in FS by each food and the subsystem for Scenario 1.

Scenario 1 evaluates a dietary shift towards the Mongolian national dietary recommendations. Under this scenario, the total GHGF decreases to 40.36 kg CO_2_-eq/kg. FFWT declines to 17.8% (7.17 kg CO_2_-eq/kg), indicating that changes in the food basket can reduce transport-related emissions. However, the FW subsystem remains the largest contributor and increases in relative share (from 49.3% to 57.6%) because waste-related emissions continue to dominate the post-consumption stage. The share attributed to FC decreases to 5.5%, potentially reflecting changes in cooking energy requirements and the composition of foods prepared at home (Figure 5).

These results recommend that combined strategies of dietary transitions on the targeted foods and waste reduction policies would yield substantially high GHG reductions, complying with the global mitigation pathways for the FS [19]. In terms of the implications for the urban FS GHGF mitigation, the findings demonstrate that the FS GHGF of UB is driven primarily by downstream stages, particularly the FW and FFWT. This differs from many global assessments, in which agricultural production dominates emissions and stresses the importance of context-specific urban FS analysis. (i) The food waste prevention, (ii) the waste management enhancement, and (iii) zero-carbon transportation could bring climate benefits to UB, especially when combined with shifts toward nutritionally adequate diets.

### 3.4. Scenario 2- Waste Reduction Enhances GHGF Drop in the Downstream of FS

The European Union (EU) aims to reach a target to reduce food waste by 30% by 2050 [20]. The European Union aims to reduce food waste by 30% by 2030 under the revised Waste Framework Directive (European Union, 2025). In this context, the present study evaluated the impact of a 30% reduction in food waste throughout the food system. The results reveal that the total GHGF decreases from the current scenario of 47.13 kg CO_2eq_/kg to 39.64 kg CO_2eq_/kg (Figure 4). This reduction is primarily driven by the food waste (FW) subsystem, whose GHGF declines to 16.33 kg CO_2eq_/kg, while the GHGF of the other subsystems remains largely unchanged. Despite the overall reduction in total emissions, the GHGF associated with the agricultural production (AFP), food processing and transformation (FP and FPT), food supply (FS), and food consumption (FC) subsystems does not differ significantly from the current scenario.

## 4. Discussion

### 4.1. Current FS Statement of Ulaanbaatar City

Annually, 1.2 million tons of solid waste are generated in Ulaanbaatar, but only 17–20% is recycled, while the rest is dumped directly into landfills, which pollute the soil. In fact, the population of Ulaanbaatar has grown sevenfold over the last 3 years, and food waste accounts for 41% of total household waste in the summer and 36.2% in the winter [21]. Traffic is another factor with environmental drawbacks, such as diesel-powered public transportation in Ulaanbaatar, resulting in significant environmental and health issues due to high levels of air pollution [21]. The quality of water resources and the drinking water supply, such as anthropogenic pressures on the urban environment, create health risks for the population due to the entry of toxic pollutants into water bodies [22]. When production is dominated by fuel emissions, the storage phase’s impact can be substantial because it relies on the city’s coal-intensive grid electricity [23]. Accurately modelling this combined subsystem requires summing the distinct inventories for each phase to avoid underestimating the local FS’s total climate impact.

Regarding the FW subsystem, it is inevitable to explain UB’s current waste management system. Currently, UB has five landfills [24], which lack complex treatment facilities or leak prevention, and the soil is compacted over the waste after it is burned [25]. However, the three largest landfills use heavy vehicles to compress open-field solid waste. To analyse the GHGF, the study conducted surveys at the landfills and estimated the GHG footprint using the heavy vehicles’ fuel consumption. The UB’s food waste studies are significantly low; thus, the field survey conducted in this study aims to outline the amount of food waste in each subsystem. There is only one proper study, done by The Asia Foundation (2019), about the food waste ratio across subsystems, and its results are close to those of this study. Based on the household and commercial surveys, food waste was managed through three distinct pathways: (i) municipal collection and landfilling (71% of total food waste mass), (ii) direct feeding to livestock or pets (18%), and (iii) open burning or uncontrolled dumping (11%). For landfilled waste, the GHGF was calculated using landfill gas models (Equation (3)). Stratified analysis by housing type reveals distinct footprints: ger district households (*n* = 630) generate 14.2 kg CO_2_-eq per capita per month from food consumption and waste, compared to 29.6 kg CO_2_-eq for apartment households (*n* = 489). The difference is driven primarily by lower food waste rates in ger areas (12% vs. 21%) and lower cold-chain reliance. However, ger district households have 35% higher transport-related emissions due to longer travel distances to markets.

The environmental impact of food in UB presents a carbon narrative that contradicts the global norm. Where worldwide assessments typically find that the AFP is the dominant source of the GHGF, this study determined that UB’s FS is decisively post-production-driven. The GHGF of 55% in Subsystem 6: FW can also be caused by Mongolia’s short growing season, year-round landfill operations, and a linear metabolic flow that terminates in high-GHG-generating waste dumps. This finding mandates a fundamental shift in climate policy, moving the focus from the pasture to the city’s final sink. When food waste is dumped in a landfill, the biogenic carbon it contains is converted into potent GHG over the course of decades. The emission represents a “double penalty”: all resources and emissions invested in producing, transporting, and processing the wasted food (captured in the other five subsystems) are rendered futile and are then compounded by a massive, direct GHG release at the disposal site. This result strongly corroborates LCA, which finds landfilling to be the most GHG-intensive waste management pathway by a significant margin [26]. Although our results are specific to Ulaanbaatar, the methodological framework—disaggregated six-subsystem LCA with primary data on local transport, energy, and waste handling—can be replicated in any city. Key transferable insights include: (i) in cold-climate cities, waste-management emissions often dominate over production; (ii) housing type (apartment vs. peri-urban) is a stronger determinant of per capita footprint than income alone; and (iii) policy interventions must align with existing waste infrastructure (e.g., landfill gas flaring is more feasible than AD in early-stage systems). Researchers adapting our approach should substitute local emission factors for electricity, heating, and landfill gas models.

### 4.2. Implication for Urban Food–Climate Policy in UB

The dominance of the FW subsystem in UB’s food system GHGF establishes a clear and actionable mitigation hierarchy: the most impactful strategy is to interrupt the current linear flow of organic waste to landfills and replace it with prevention, improved handling, and lower-emission end-of-life management. This can be achieved by prioritising source reduction through public awareness campaigns on proper food storage and portioning, together with improved inventory management in retail, which can reduce the volume entering the waste stream [27]. Implementing landfill gas capture as an immediate interim measure at existing sites to mitigate a significant portion of methane emissions should be considered [28]. Strengthening mid-system resilience—especially for high-impact products such as meat and dairy that constitute a large fraction of UB’s waste mass and have high methane potential—by improving logistics and cold-chain storage, which reduces both upstream sunk emissions and downstream landfill releases, should also be considered [29]. The integration of food-system interventions with broader energy system decarbonisation, given Mongolia’s carbon-intensive grid, by promoting highly energy-efficient measures and deploying options such as anaerobic digestion that can generate recoverable energy, coordinated with renewable energy and transport electrification policies to ensure real carbon savings. The anaerobic digestion of organic waste in cold climates can achieve net negative emissions when heat is recovered [30]. Mongolia’s Green Development Policy (2014) targets a 20% reduction in municipal waste landfilling by 2025, but does not specify organic waste separately. Ulaanbaatar’s Master Plan 2030 includes a new waste-to-energy facility and improved transfer stations.

### 4.3. The Limited Role of Dietary Change in This Structure

A move toward the national dietary shift can reduce ruminant meat and increase poultry and vegetables, which would lower the GHGF of the food basket itself, as ruminant meat has a vastly higher production footprint [7,31]. Our scenario modelling indicates this could reduce the total system GHG by 11%. In other words, the dominance of the FW subsystem remains unchallenged, with landfilled waste still contributing about half of the remaining emissions, and it is a critical finding that dietary guidance benefits public health and reduces agricultural pressures. The systemic flaw lies not just in what is eaten, but in what is wasted and how that waste is managed. Also, improving the carbon efficiency of existing meat production through better pasture management and shorter transport-to-slaughter distances can reduce the production-phase GHGF by an estimated 18–25% without changing intake.

The Monte Carlo simulation with 10,000 iterations reveals that both circular economy interventions achieve significant emission reductions (*p* < 0.001). Food waste reduction, framed as a resource efficiency strategy, demonstrates superior effectiveness with 15.9% mean reduction (95% CI: 13.4–18.4%), compared to 10.0% (8.2–11.9%) for the dietary shift. Waste reduction shows 99.9% probability of outperforming the dietary shift, supporting circular economy prioritisation. Subsystem analysis identifies food waste (CV = 18.6%) as the primary uncertainty driver, highlighting opportunities for improved waste valorisation. These findings align with the EU circular economy targets and support evidence-based resource management policies.

### 4.4. Limitation

Emission factors of subsystems, particularly AFP (400–600 g CO_2_eq/kg for plant-based foods) and transportation, rely on international lifecycle assessment databases and meta-analyses [18,32]. Regarding this study, a lack of qualified analysis and database information about the UB GHGF can lead to uncertainty, as local practices in livestock husbandry, soil management, and logistics might be different from the global averages. In this study, the estimation does not consider the CH_4_ emission from the soil in the landfill due to the current situation of the landfills of UB, as UB only has conventional landfills. As CH_4_ from landfill soil contributes high amounts of carbon to the environment, it should be studied further in the next research studies to quantify the full GHGF of the whole food system. Even though the study quantifies GHG emissions, it does not assess other critical sustainability dimensions, such as water and land use changes, air pollution (beyond GHGs), or socio-economic impacts. A full sustainability appraisal would be needed to avoid burden shifting and to evaluate trade-offs, such as the economic impact on herders from reduced red meat consumption.

Finally, the analysis and proposed interventions are techno-economic in nature. They do not fully address the behavioural, cultural, and institutional barriers to implementation. The success of waste segregation, dietary shifts, and acceptance of new technologies like AD plants depends on social factors that require dedicated interdisciplinary research. This analysis is limited by reliance on literature-based uncertainty ranges rather than empirical waste characterisation data. While 10,000 Monte Carlo iterations ensure statistical convergence, correlations among circular economy interventions were not modelled, potentially underestimating synergistic effects. The analysis excludes downstream resource recovery pathways and industrial symbiosis opportunities. Future research should incorporate material flow analysis and the dynamic modelling of circular food system transitions with empirical uncertainty quantification.

## 5. Conclusions

UB’s FS GHGF is defined by its endpoint. The findings obtained indicate that almost half of its GHG originates from the food waste subsystem and fundamentally reorients the city’s climate challenge from one of agricultural production to one of urban waste metabolism. This downstream dominance is a direct consequence of a system where continuous landfill operations eclipse seasonal agricultural activity, transforming organic waste into a leading source of atmospheric methane. The policy imperative is therefore unambiguous: the most powerful lever for rapid decarbonisation is the creation of a circular organic waste loop. By prioritising waste prevention, mandating diversion to anaerobic digestion, and capturing landfill gas, UB can directly attack its largest emission source. This strategy simultaneously addresses climate change, energy security, and air pollution. While improvements in agricultural efficiency and dietary patterns remain worthwhile, they are secondary to the urgent task of transforming the landfill from a climate liability into a resource hub. In FS decarbonisation, the map is not the territory. Local metabolic flows—shaped by climate, infrastructure, and practice—can create emission profiles that defy global averages. Effective climate action must begin with a clear-eyed, local diagnosis. In Ulaanbaatar, that diagnosis points unequivocally to the landfill, making its transformation the cornerstone of a sustainable urban food future.

“To ensure political and institutional feasibility, we rank interventions by readiness (based on stakeholder interviews with UB waste department officials, January 2024):

• High feasibility, low cost: public awareness on reducing meat waste (already in city budget), landfill gas flaring at the main site (feasible under existing operator’s license).
• Medium feasibility, medium cost: source-separated organic waste collection in apartment blocks (pilot underway in Bayanzurkh district).
• Low feasibility, high cost: centralised anaerobic digestion (requires new capital investment and policy changes).

We therefore recommend starting with the high-feasibility actions and building toward AD only after institutional capacity is strengthened.”

## Data Availability

The original contributions presented in this study are included in the article. Further inquiries can be directed to the corresponding author.

## Abbreviations

The following abbreviations are used in this manuscript:

AFP	Agricultural Food Production
CH_4_	Methane
CO_2_	Carbon Dioxide
CO_2eq_	Carbon Dioxide Equivalent
EU	European Union
FC	Food Consumer
FR	Food Retailer
FP and FS	Food Producer and Food Storage
FS	Food System
FFWT	Food and Food Waste Transportation
FW	Food Waste
GHG	Greenhouse Gas
GHGF	Greenhouse Gas Footprint
GTP100	Global Temperature Change Potential Over a 100-Year Time Horizon
IPCC	Intergovernmental Panel on Climate Change
LCA	Life Cycle Assessment
N_2_O	Nitrous Oxide
SA	Scenario Analysis
UB	Ulaanbaatar
